# Historic emissions from deforestation and forest degradation in Mato Grosso, Brazil: 1) source data uncertainties

**DOI:** 10.1186/1750-0680-6-18

**Published:** 2011-12-30

**Authors:** Douglas C Morton, Marcio H Sales, Carlos M Souza, Bronson Griscom

**Affiliations:** 1 NASA Goddard Space Flight Center, Greenbelt MD USA; 2 Instituto do Homem e Meio Ambiente da Amazônia (Imazon), Belém, PA, Brazil; 3 The Nature Conservancy, Arlington, VA USA

**Keywords:** Amazon, REDD+, IPCC, Tier, Approach, Landsat

## Abstract

**Background:**

Historic carbon emissions are an important foundation for proposed efforts to Reduce Emissions from Deforestation and forest Degradation and enhance forest carbon stocks through conservation and sustainable forest management (REDD+). The level of uncertainty in historic carbon emissions estimates is also critical for REDD+, since high uncertainties could limit climate benefits from credited mitigation actions. Here, we analyzed source data uncertainties based on the range of available deforestation, forest degradation, and forest carbon stock estimates for the Brazilian state of Mato Grosso during 1990-2008.

**Results:**

Deforestation estimates showed good agreement for multi-year periods of increasing and decreasing deforestation during the study period. However, annual deforestation rates differed by > 20% in more than half of the years between 1997-2008, even for products based on similar input data. Tier 2 estimates of average forest carbon stocks varied between 99-192 Mg C ha^-1^, with greatest differences in northwest Mato Grosso. Carbon stocks in deforested areas increased over the study period, yet this increasing trend in deforested biomass was smaller than the difference among carbon stock datasets for these areas.

**Conclusions:**

Estimates of source data uncertainties are essential for REDD+. Patterns of spatial and temporal disagreement among available data products provide a roadmap for future efforts to reduce source data uncertainties for estimates of historic forest carbon emissions. Specifically, regions with large discrepancies in available estimates of both deforestation and forest carbon stocks are priority areas for evaluating and improving existing estimates. Full carbon accounting for REDD+ will also require filling data gaps, including forest degradation and secondary forest, with annual data on all forest transitions.

## 1. Background

Tropical deforestation accounted for approximately 12% of anthropogenic CO_2 _emissions in 2008 [1]. Forest degradation from fire, logging, and fuel wood collection represents an additional source of carbon emissions from land use activities in tropical forest regions [1-6]. Recognition of the important contributions from deforestation and forest degradation to anthropogenic greenhouse gas emissions led to proposals for Reduced Emissions from Deforestation and forest Degradation (REDD) to be included in a post-2012 climate agreement under the United Nations Framework Convention on Climate Change [7]. In 2010, the Cancun Agreements expanded the scope for climate mitigation activities in forests to include the conservation and enhancement of forest carbon stocks and sustainable forest management, or REDD+ [8].

Proposed REDD+ mechanisms require a baseline or reference emissions level against which future emissions can be compared [9,10]. Previous scientific studies have estimated historic deforestation carbon emissions at pan-tropical [1,3,11-13] or regional spatial scales, such as the Brazilian Amazon [14-19]. However, the spatial and temporal resolutions of previous deforestation emissions estimates are likely too coarse for national REDD+ baselines, given the potential inclusion of sub-national activities [8]. In addition, the input data and methods in these studies were not necessarily consistent with guidance on national-scale reporting of emissions from forest lands from the Intergovernmental Panel on Climate Change [20,21]. A range of forest carbon stock and deforestation data products exist at national and sub-national scales that could be used to establish historic emission levels [21], but the suitability of existing data for estimating historic carbon emissions and associated uncertainties has not been thoroughly evaluated.

The level of uncertainty in historic emissions baselines is critical for REDD+. Uncertainty in forest carbon emissions arises from estimated rates of deforestation and forest degradation, forest carbon stocks [22,23], and emissions factors [16,24,25]. Large uncertainties could undermine the effectiveness of REDD+ by limiting the ability to generate credits from mitigation actions, especially if a conservative approach is used to estimate REDD+ credits [26,27]. In the absence of a conservative approach, large uncertainties in historic emissions could lead to a situation in which mitigation actions fail to generate climate benefits (i.e., "hot air").

Research to reduce uncertainties in REDD+ baselines at national or sub-national scales may generate both scientific and policy payoffs. Tropical deforestation remains the most uncertain term in the global carbon budget [28]. Attention to the source and magnitude of uncertainties in emissions estimates at the national level can therefore help to constrain the global carbon balance. Reducing uncertainties at the national level may remove potential discounts from REDD+ carbon credits. Recent studies suggest that uncertainties in rates of deforestation and forest degradation [26,29] and forest carbon stocks [30] can dramatically alter the cost-benefit calculation for REDD+ from the country perspective.

Here, we use a structured approach to evaluate the source and magnitude of uncertainties in historic forest carbon emissions for the Brazilian State of Mato Grosso. Mato Grosso is a hotspot of recent deforestation, accounting for more than 15% of humid tropical forest losses worldwide during 2001-2005 [31-33]. Compared to other tropical forest regions, Mato Grosso also has a wealth of data with which to evaluate historic emissions. In this, the first of two research articles, we review the available data for Mato Grosso on deforestation, forest degradation, and forest carbon stocks to identify important data gaps and research needs to reduce source data uncertainties in historic forest carbon emissions estimates. We concentrate on five annual deforestation datasets and six estimates of forest carbon stocks in Mato Grosso (see Sections 5.3 and 5.4). We conclude this study with a roadmap for research in support of REDD+ based on the spatial and temporal patterns of disagreement among available data products. In the second manuscript, we describe a new model, the Carbon Emissions Simulator, to quantify the contribution from source data evaluated in this study and model parameters to total uncertainties in forest carbon emissions. The Carbon Emissions Simulator uses both Monte Carlo and error propagation techniques to quantify uncertainties in deforestation carbon emissions. By separating data and model-based uncertainties, the Carbon Emissions Simulator can be used to evaluate tradeoffs for improving historic emissions estimates by year, region, and source term. Together, these papers provide a comprehensive look at the data and research methods needed to quantify and reduce uncertainties in historic forest carbon emissions estimates for REDD+.

## 2. Results

### 2.1 Deforestation

**Figure 1:**
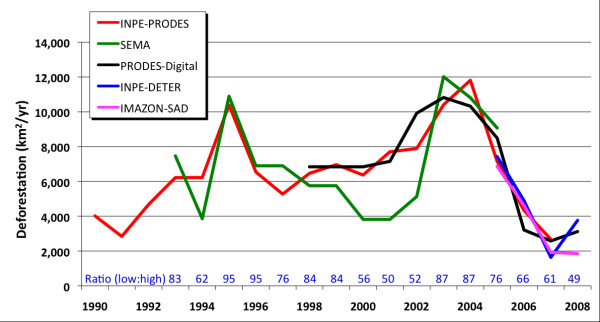
**Annual deforestation in Mato Grosso State during 1990-2008**. Blue numbers indicate the ratio between low and high annual deforestation estimates. Individual data products are described in Table 1.

All five deforestation data products identified periods of increasing (2001-2004) and decreasing (2005-2007) deforestation rates in Mato Grosso (Figure 1, Table 1). Annual deforestation rates were highly variable during the study period, ranging between 2,203 and 11,082 km^2 ^yr^-1 ^during 1990-2008. Three years with deforestation rates greater than 10,000 km^2 ^yr^-1 ^accounted for more than 25% of the total forest loss during this period (1995, 2003, and 2004).

**Table 1:** Data sources for historic deforestation in Mato Grosso, Brazil.

Dataset	Approach	Temporal Coverage	MMU ^1^	Reference	Sensor	Method
INPE-PRODES	2	1987-2008	6.25 ha	[58]	Landsat	Single image, visual interpretation
PRODES-Digital **^2^**	3	1997-2008	1 ha	[33]	Landsat	Single image, digital processing, visual interpretation
SEMA **^2^**	3	1992-2005	1 ha	[32]	Landsat	Single image, digital processing, visual interpretation
IMAZON-SAD ^3^	3	2005-2008	12.5 ha	[59]	MODIS^2^	Two images, digital processing, automated analysis
INPE-DETER ^3^	3	2004-2008	25 ha	[60]	MODIS^2^	Single image, digital processing, visual interpretation

**^1 ^**Minimum mapping unit (MMU): the smallest area of new deforestation identified in any year.

^2 ^Annual deforestation estimates were not available from PRODES-Digital during 1997-2000 or SEMA for 1996, 1998, and 2000. Average values of forest loss between image dates were used in these years (e.g., forest area change between 1995-1997 images was divided equally between 1996 and 1997).

**^3 ^**Alert data products provide near-real time monitoring of deforestation using imagery from the MODIS sensors at 250 m resolution. These data are primarily intended to identify the location of new deforestation, especially for deforestation events > 25 ha, rather than provide robust estimates of forest area change.

On an annual basis, deforestation rates from different data products exhibited considerable variability (Figure 1). In three consecutive years (2000-2002), deforestation rates from SEMA were approximately half those from PRODES-Digital, despite reliance on similar Landsat base data for both products. The range of annual deforestation rates exceeded the expected performance of satellite-based approaches (80-95% accuracy, [34]) in more than half of the years with multiple satellite-based deforestation products (1998-2008). Using a confidence interval of ± 20%, low and high estimates of annual deforestation did not overlap in these six years. The inclusion of MODIS-based deforestation data increased the range of annual deforestation estimates in 2005-2008, yet the INPE-DETER and Imazon-SAD estimates only represented the high and low values in 2008.

**Figure 2:**
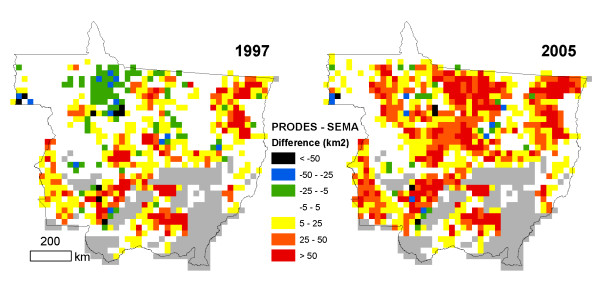
**Spatial differences between PRODES-Digital and SEMA estimates of cumulative deforestation through 1997 (a) and 2005 (b), summarized as the difference in remaining forest area (km^2^) within each 0.25° cell**. Areas outside of the PRODES forest mask appear gray.

The legacy of differences in satellite-based deforestation datasets can also be seen in the spatial distribution of cumulative forest loss (Figure 2). In 1997, SEMA deforestation estimates indicated greater cumulative forest losses than PRODES-Digital data in northern Mato Grosso (Figure 2a). By 2005, cumulative forest losses derived from PRODES-Digital data were higher across the state, with the greatest differences around the Xingu River basin in eastern Mato Grosso (Figure 2b). These areas of greatest uncertainty highlight the need for additional field and remote sensing research to identify the causes of consistent spatial discrepancies among satellite-based estimates of forest area change.

### 2.2 Forest Carbon Stocks

Average forest carbon stock estimates from Tier 1 and Tier 2 data products for Mato Grosso varied by a factor of two (Table 2). The Tier 1 estimate of forest carbon stocks in Mato Grosso was the highest estimate of total carbon (206 Mg C ha), based on the value for humid tropical forests in South America. The Tier 1 root-shoot ratio (0.37) was much higher than for other products (0.21-0.26), suggesting that below ground biomass (BGB) accounts for part of the difference between Tier 1 and Tier 2 carbon stock estimates. Among Tier 2 data products, the source of plot data, number of parameters in the biomass expansion factor, and methods to interpolate between plot locations all contributed to the difference in carbon stock estimates. The wide range of average forest carbon stocks for Mato Grosso suggests that per-product uncertainty could be greater than ± 50%, similar to an earlier assessment of biomass data products by [22].

**Table 2:** Tier 1 and Tier 2 data sources for tropical rainforest carbon stocks in Mato Grosso.

Source	Total C: AGLB+AGDB+BGB (Mg C/ha) ^1^	AGDB, BGB (% AGLB)	Plot Data	Carbon Fraction (CF)	Tier ^2^
IPCC **^3^**	SA: 206	9%, 37%	N/A	0.47	1
Houghton *et al. *2001	BA: 192	9%, 21%	Literature Review	0.5	2.a
Brown & Lugo 1992	BA: 156	9%, 21%	RADAM ^4^	0.5	2.a
Nogueira *et al. *2009	MT: 159.7	13.91%, 25.8% **^5^**	RADAM ^4^	0.485	2.a
Imazon; Sales *et al. *2007	MT: 130.4 ± 44.8	13.91%, 25.8% **^5^**	RADAM ^4^	0.485	2.m
Saatchi *et al. *2007	MT: 99.0 ± 58.0	9%, 21%	Houghton *et al. *2001	0.5	2.m

Tier 1 data are the IPCC default values for forest carbon stocks, whereas Tier 2 indicates country-specific data (see Table 4). Total forest carbon (C) was estimated from aboveground live biomass (AGLB) using conversion factors from each source for aboveground dead biomass (AGDB) and below-ground biomass (BGB) as a percentage of AGLB.

**^1 ^**Average total carbon in forest biomass for tropical rainforest South America (SA), Brazilian Amazon (BA), or Mato Grosso (MT).

**^2 ^**Tier 2 biomass data products were divided between regional or state-wide average values (Tier 2.a) and spatially-explicit maps of forest biomass (Tier 2.m).

**^3 ^**As reported by [80]

**^4 ^**The RADAMBRASIL floristic inventory (DPNM, 1973-1983).

**^5 ^**Nogueira et al (2009) applied additional correction factors for AGLB in dense (10.5%) and non-dense (15.7%) forest types. These factors were also included in the Imazon product.

**Figure 3:**
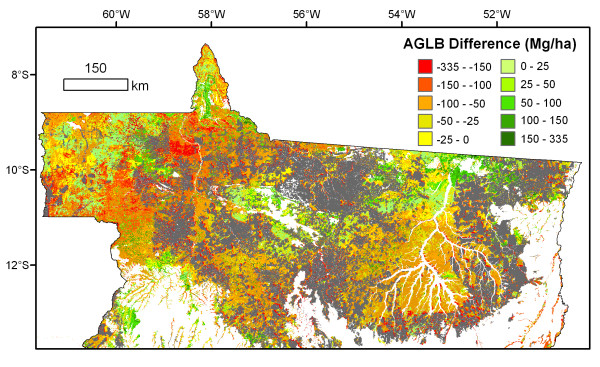
**Map of differences between Saatchi *et al***. [75] and Imazon [76] estimates of AGLB in northern Mato Grosso state. Imazon estimates exceed those of Saatchi *et al. *in red, orange, and yellow areas, while green areas indicate higher AGLB estimates from Saatchi *et al. *Deforestation through 2005 is shown in gray, and non-forest areas within Mato Grosso appear white. Individual data products are described in Table 2.

The spatial distribution of forest carbon stocks in Mato Grosso differed markedly between Tier 2.m data products considered in this study. The Saatchi *et al. *and Imazon estimates of aboveground live biomass (AGLB) disagreed by ~50 Mg ha^-1 ^in central and eastern portions of the state, and differences between the two products exceeded 100 Mg ha^-1 ^in northwest Mato Grosso (Figure 3). The conversion from AGLB to total biomass amplified the spatial discrepancies in Figure 3 because expansion factors for BGB and aboveground dead biomass (AGDB) for the Imazon product were larger than in the Saatchi *et al. *data product (see Table 2).

### 2.3 Deforested Biomass

Deforestation in Mato Grosso during 1993-2008 was concentrated in low biomass forest types. Average biomass in deforested regions increased during the study period (Table 3) but remained below state-wide averages (Table 2). For the combination of SEMA deforestation data with Imazon biomass estimates, average AGLB in deforested regions increased by 2.3 Mg ha^-1 ^yr^-1 ^during 1993-2005 (R^2 ^= 0.85, Table 3). PRODES-Digital data also suggest an increasing trend in average AGLB in deforested areas using Imazon biomass estimates during 2001-2008 (1.8 Mg ha^-1^yr^-1^, R^2 ^= 0.43). Differences in the location of recent deforestation between PRODES-Digital and SEMA had little impact on the average biomass in deforested areas during 2001-2005 from the IMAZON product (< 2 Mg ha^-1^), as discrepancies between these products were widely distributed across low and high biomass forests in Mato Grosso by 2005 (Figure 2b).

**Table 3:** Mean aboveground live biomass ± 1 SD in areas of recent deforestation (Mg ha^-^^1^).

Year	SEMA/Imazon	PRODES-Digital/Imazon	**PRODES-Digital/Saatchi ***et al*.
1993	158.9 ± 46.9		
1994	152.6 ± 43.2		
1995	167.0 ± 54.9		
1996	165.5 ± 49.5		
1997	165.5 ± 49.5		
1998	174.4 ± 61.0	180.5 ± 55.9	
1999	174.4 ± 61.0	180.5 ± 55.9	
2000	179.4 ± 56.2	180.5 ± 55.9	
2001	179.4 ± 56.2	166.6 ± 54.1	
2002	181.1 ± 53.4	172.7 ± 57.0	
2003	179.6 ± 56.9	183.4 ± 58.8	
2004	185.7 ± 59.0	181.0 ± 56.5	
2005	180.8 ± 57.6	184.6 ± 61.4	135.5 ± 87.3
2006		179.8 ± 63.7	126.7 ± 90.5
2007		187.0 ± 67.8	143.8 ± 90.2
2008		179.1 ± 56.0	136.5 ± 82.3

Tables 1 and 2 provide additional details regarding Tier 2.m biomass data products (Imazon, Saatchi *et al*.) and Approach 3 deforestation products (SEMA, PRODES-Digital), respectively.

Overall, the choice of Tier 2.m biomass data had a larger impact on estimates of deforested biomass than the trend of increasing biomass in recently deforested areas from either product. The difference in average AGLB between Imazon and Saatchi *et al. *data products for deforestation during 2005-2008 (47.8 Mg ha^-1^) was larger than the total increase in average deforested biomass from either product during the study period (< 30 Mg ha^-1^, Table 3).

## 3. Discussion

The range of available deforestation and biomass data products provides a first estimate of source-data uncertainties in historic deforestation carbon emissions. Findings in this study highlight how specific years, regions, and data products contribute to potential variability in deforestation emissions estimates. Large (25-50%) discrepancies remain between estimates of forest carbon stocks and annual deforestation from different data products, even for estimates at the same Tier or Approach. Reconciling these differences is essential to reduce uncertainties in historic deforestation emissions estimates and prevent the propagation of errors from subsequent land-use transitions in disputed areas.

Reducing source data uncertainties requires careful methods to substitute space for time. The archive of Landsat satellite imagery is a rich resource for countries interested in revising estimates of forest area changes from 1972-present [35]. Landsat resolution (30 m) is suitable for detailed estimates of forest area change [34], provided that an accuracy assessment can be conducted using very high resolution (< 5 m) imagery from airborne or satellite data sources [36]. In the case of Mato Grosso, where most deforestation occurs in large clearings (> 25 ha, [37]), forest area change estimates from moderate resolution (250 m) deforestation monitoring systems do not differ much from estimates obtained from Landsat-based deforestation maps (see Figure 1, Table 1). However, deforestation alert systems are inappropriate for monitoring small forest clearings [38] or forest degradation from selective logging [39] for estimates of historic carbon emissions.

In contrast to the rich archive of historic satellite data, there is limited historic forest inventory data for Mato Grosso. Improving estimates of tropical forest carbon stocks will therefore require new data collection. A new National Forest Inventory is already underway in Brazil (http://ifn.florestal.gov.br), with field plots distributed on a regular grid (20 km × 20 km). New technologies offer the possibility to generate spatially explicit biomass maps using a more limited network of forest inventory plots and large-area sampling of forest heights with airborne or spaceborne LiDAR [40-42]. However, contemporary estimates of forest carbon stocks at the deforestation frontier must then be paired with data on historic deforestation and forest degradation to account for the impacts of historic land use on contemporary measurements (e.g., [43]). Routine sampling may be needed to maintain updated field or LiDAR-based information on forest carbon stocks for REDD+ [44] because static reference data are unable to account for increases in forest carbon stocks over time (e.g., [45]) or reductions in biomass from forest disturbance (e.g., [46,47]).

**Figure 4:**
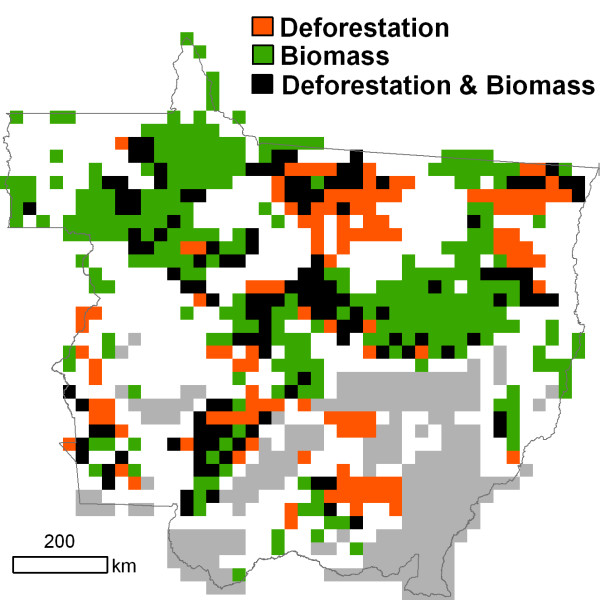
**Data needs to reduce uncertainties in historic deforestation carbon emissions from Mato Grosso, summarized at 0.25° spatial resolution**. White cells indicate areas where Landsat-based estimates of cumulative deforestation through 2005 differ by > 40 km^2^. Gray cells indicate regions where average Tier 2.m estimates of AGLB in remaining forest in 2005 differ by > 50 Mg ha^-1^. Cells with data needs for both deforestation and biomass appear black.

**Figure 5:**
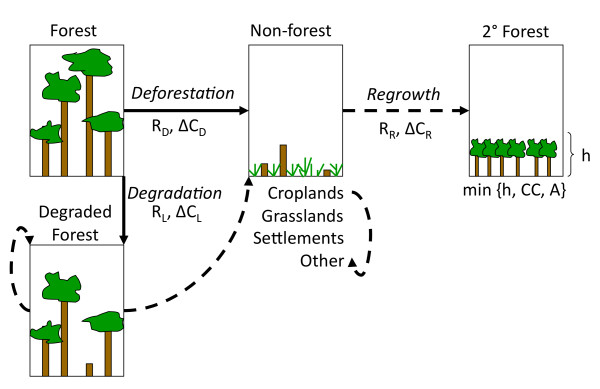
**Land use transitions and related data needs to estimate carbon emissions from deforestation and forest degradation**. Full carbon accounting requires data for the rate (R) of area change and associated changes in carbon stocks (ΔC) for deforestation (D), forest degradation (L), and regrowth (R). All forest lands must meet minimum height (h), crown cover (CC), and area (A) requirements, according to each country's national forest definition. Solid arrows represent primary transitions from forest to non-forest or degraded forest lands; dashed arrows represent secondary land-use transitions.

What research is needed to reduce source data uncertainties in Mato Grosso and other Amazon regions? New measurements of forest carbon stocks and new estimates of forest area changes from remotely-sensed data are most critical in regions where existing products disagree (Figure 4). Areas with high uncertainties in both forest biomass and deforestation rates provide an opportunity to collect complementary information on land use and carbon stocks to improve estimates of historic carbon emissions. Improved estimates of forest carbon stocks in areas with concentrated historic deforestation are a specific priority for efforts to quantify historic emissions and establish REDD+ baselines. Additional data collection and analysis in these areas are needed to develop a consistent, validated approach for full carbon accounting from deforestation and forest degradation (Figure 5).

At least two factors likely contributed to the observed spatial and temporal discrepancies in annual deforestation rates for Mato Grosso. First, none of the satellite-based deforestation estimates were developed specifically for REDD+. As a result, forest degradation from logging and fire may have been included in historic deforestation estimates, especially in years with extensive damages from understory forest fires [48]. Incomplete information on forest degradation and secondary forest dynamics also contributes to source data uncertainties for estimating net forest carbon emissions in Mato Grosso. Full carbon accounting from deforestation and forest degradation will require careful consideration of sequential land-use transitions (Figure 5). A time-series approach to track deforestation, degradation, and secondary forest dynamics using annual satellite imagery could improve emissions estimates for Mato Grosso and other tropical forest regions by reducing misclassification and "double counting" errors [48] that occur when degraded forests are deforested for agricultural use [49]. Second, time series methods may also improve the consistency of deforestation estimates over time. Deforestation estimates in this study were based on interpretation of a single satellite image or a comparison between two successive images. Time series methods that consider longer periods of disturbance and recovery may improve the accuracy of change detection [50], especially for retrospective analyses to establish historic baselines. Annual satellite data can be used to confirm continued agricultural use of previously deforested areas, forest recovery following degradation, and the age of secondary forests from land abandonment to improve carbon stock estimates in areas of active land use change.

In addition to reducing source data uncertainties, reanalysis of historic changes in forest area can also facilitate sub-national allocation of deforestation baselines. Brazil recently selected the 1996-2005 period for deforestation baseline calculations [51]. However, annual PRODES-Digital deforestation data are only available beginning in 2000. Allocation of baseline deforestation information to Amazon states can be accomplished using PRODES statistics (Approach 2), but below the state scale, regional or project-scale activities may require a new analysis of historic deforestation and forest degradation to provide Approach 3 data for all years during the baseline period.

The range of available data products provides an indication of the spatial and temporal variability associated with estimates of deforestation and forest carbon stocks. However, total uncertainties in historic emissions cannot be estimated without validation efforts to characterize per-product uncertainties. Validation needs are greatest in areas where existing products disagree (Figure 4), but all deforestation and carbon stock data products should include a robust validation plan with routine field measurements and airborne or spaceborne very high resolution imagery (< 5 m).

Given the need for routine data collection on forest transitions and associated carbon losses, development and maintenance of reporting information for REDD+ will likely require dedicated capacity for satellite and field data analysis. Consistent methods for data analysis are also critical for REDD+ [27]. Even in a well-characterized region such as Mato Grosso, multiple deforestation data products were required to consider forest area changes during 1990-2008 because no single product provided annual estimates during the entire study period. The development of standards for REDD+ monitoring, reporting, and verification (MRV) provides an opportunity to design a system that can lower uncertainties in emissions estimates over time using the comparative approach described in this paper. Ideally, the analysis in this study would be the first iteration of a routine process to target new data collection in regions and years with largest uncertainties in carbon stock and deforestation estimates.

## 4. Conclusions

This study reviewed available data products for deforestation, forest degradation, and forest carbon stocks in Mato Grosso, Brazil to assess the level of uncertainty in source data for estimating historic forest carbon emissions for REDD+. Deforestation data showed considerable spatial and temporal variability, with Landsat-based estimates of annual deforestation differing by > 20% in most years. Forest carbon stock estimates exhibited even greater variability, with more than a two-fold difference in carbon stock estimates in northwest Mato Grosso. Limited information was available on forest degradation and secondary forest regeneration, suggesting that full carbon accounting for REDD+ cannot be achieved without additional satellite data analysis to quantify annual transitions involving degraded or regenerating forests.

The diversity of deforestation and carbon stock estimates for Mato Grosso provides an initial indication of research needs to address source data uncertainties for REDD+. Spatial and temporal patterns of disagreement show priority areas for new data collection, and a coordinated strategy to estimate forest carbon stocks and validate deforestation estimates in these areas could target the main source data uncertainties in Mato Grosso. Data needs for REDD+ differ from previous uses of deforestation information for enforcement of environment laws and private property rights. The additional focus on source data uncertainties for REDD+ could reduce large uncertainties in current emissions estimates, thereby increasing the likelihood of generating benefits from REDD+ actions.

## 5. Materials and methods

Below, we synthesize relevant IPCC guidance for source data on changes in the area and carbon stocks in forest lands (Section 5.1), describe the Mato Grosso study area (Section 5.2), and review available data on forest area changes (Section 5.3) and carbon stocks (Section 5.4).

### 5.1 IPCC Tiers and Approaches

The definition of 'forest' forms the foundation of REDD+ and related initiatives, establishing the spatial extent of forest cover and the criteria for deforestation. The Brazilian government defines their forest land as areas of at least one hectare in size with more than 30% crown cover of trees ≥ 5 m in height. This definition selects the upper end of ranges for area (0.04-1.0 ha), crown cover (10-30%), and tree height (2-5 m) in guidelines established by the UNFCCC for the Clean Development Mechanism of the Kyoto Protocol [52]. Deforestation occurs when any of these thresholds are crossed, typically during the conversion of forest for agricultural use (Figure 5). Within the scope of REDD+, forest degradation is generally considered a reduction in carbon stocks within forest land remaining as forest [21], although a precise definition of forest degradation has not been adopted [53].

Data on the rates of forest transitions and associated changes in carbon stocks are classified according to the methods used for data collection (Table 4). We followed this guidance when reviewing and analyzing available forest area change data (Activity Data) and data on changes in carbon stocks (Emissions Factors) from transitions between forest land and other land uses [21]. For estimates of deforestation, moving from Approach 1 to Approach 3 area change data involves a shift from global or national survey methods (e.g., the Food and Agricultural Organization's periodic Forest Resource Assessment surveys) to spatially-explicit estimates from satellite remote sensing data (Table 4). Approach 3 data are recommended as the basis for establishing REDD+ baselines [21], since fine-scale spatial information (20-60 m) is necessary to track sequential land-use transitions at a given location through time (Figure 5). The specificity of source data on forest carbon stocks also increases from continental-scale averages for each forest type (Tier 1) to country-specific information (Tier 2). Few countries have established Tier 3 efforts to repeatedly measure or model forest carbon stocks that could be used to estimate historic emissions.

**Table 4:** Summary of IPCC data categories for Activity Data on forest area changes and Emission Factors for changes in carbon stocks from deforestation and forest degradation.

Approaches for Activity Data: Forest Area Changes	Tiers for Emission Factors: Changes in Carbon Stocks
1. Non-spatial country statistics	1. IPCC default values by continent and forest type
2. Maps, surveys, and other national statistical data	2. Country specific data for key factors
3.Spatially explicit data from interpretation of remote sensing imagery	3.National inventory of carbon stocks, via repeated measurements of key stocks through time or modeling

Approach 1 and Tier 3 data products were unavailable for Mato Grosso during 1990-2008. Please see [21] for a more complete discussion of IPCC Good Practice Guidance.

### 5.2 Study area

**Figure 6:**
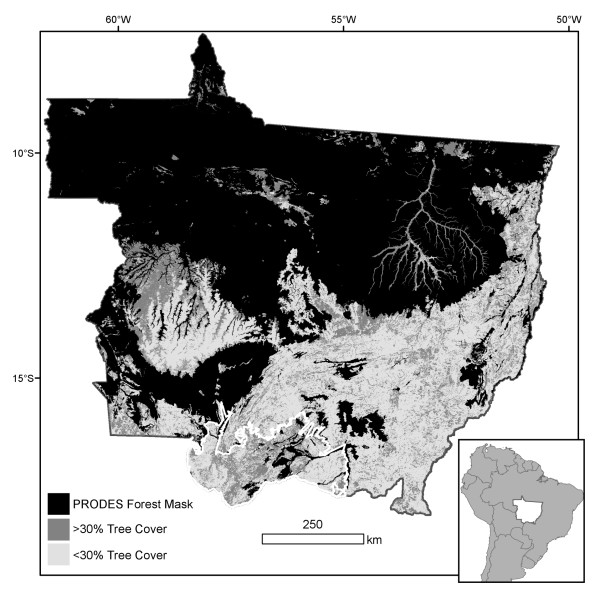
**Forest cover extent (black) from the INPE PRODES program in the Brazilian State of Mato Grosso (inset, white)**. Outside of the PRODES forest mask, areas with > 30% tree cover in 2001 appear dark gray [55]. The Pantanal biome in southern Mato Grosso is outlined in white.

The state of Mato Grosso includes the southernmost extent of Amazon forests in Brazil (Figure 6). Data from the RADAMBRASIL floristic surveys (1973-1983) indicate that Amazon forest and transition forest types initially covered two-thirds of the state [54]. The Brazilian Instituto Nacional de Pesquisas Espaciais (INPE) further refined the extent of Amazon forests using Landsat satellite data under the PRODES (Monitoramento da Floresta Amazônica Brasileira por Satélite) program of annual deforestation assessments in the Brazilian Amazon [33]. The PRODES forest mask is a common reference for many Amazon deforestation products. We adopted the PRODES forest mask for our analysis to maintain consistency with results from other studies (Figure 2); however, the PRODES mask does not include all areas that could be classified as forest in Mato Grosso based on the 30% crown cover threshold [55].

### 5.3 Forest area change data for Mato Grosso

#### 5.3.1 Deforestation

Deforestation in the Brazilian Amazon has been monitored for more than two decades using a variety of satellite sensors (e.g., [33,37,56]. Fine-scale mapping efforts have relied on Landsat or other high-resolution data (≤ 30 m pixel size), and the minimum mapping unit from these products is consistent with the one-hectare threshold for individual forest patches in Brazil's national forest definition [34]. Most studies report gross rather than net deforestation, as transitions involving secondary forest (e.g., agricultural abandonment and re-clearing) were not routinely identified in historic assessments (see Figure 5).

We evaluated annual satellite-based estimates of deforestation in Mato Grosso beginning in 1990, and we extended our evaluation through 2008 to include new deforestation products that were developed based on moderate resolution (250 m) satellite imagery. We identified five satellite-based estimates of Amazon deforestation in Mato Grosso covering part or all of the 1990-2008 timeframe (Table 1). We limited our review to annual deforestation estimates, thereby excluding available data from regional and global deforestation products with periodic (5-10 year) evaluation periods [3,11,31,56,57].

Deforestation data products were grouped according to IPCC Approach. We categorized annual deforestation statistics from the PRODES-Analog product (INPE) as an Approach 2 dataset since these data were not spatially explicit [58]. The PRODES-Digital and SEMA data products provided the longest time series of Approach 3 deforestation data. The length of the deforestation data record is critical for estimating annual emissions; a minimum of 10 years of historic deforestation data are recommended to estimate the contribution from previous clearing activity to emissions in any given year [16]. We also included two "alert" data products (INPE-DETER and Imazon-SAD) from efforts to monitor deforestation in near-real time based on moderate resolution (250 m) satellite data [59,60]. Inclusion of alert data products allowed us to characterize the additional uncertainty in annual deforestation estimates that could arise if only alert-type data on area change were available.

#### 5.3.2 Forest Degradation and Secondary Forests

Few satellite-based estimates of forest degradation exist for Mato Grosso. New algorithms to detect selective logging [4,61,62] and understory forest fires [48,61,63,64] using Landsat data were developed in Mato Grosso. However, only one estimate of selective logging was available with statewide coverage over multiple years [4]. [4] estimated that selective logging in Mato Grosso averaged 9,367 km^2 ^yr^-1 ^during 1999-2002. Excluding logged areas that were deforested by 2004, the average annual logged area during 1999-2002 was 6,923 km^2 ^yr^-1 ^[49]. No satellite-based estimates of understory forest fires or fuel wood collection were available for Mato Grosso, even for a single year.

Knowledge of the extent and frequency of land abandonment to secondary forest is critical for estimating net carbon emissions from deforestation, since carbon accumulation in secondary forests may partially offset deforestation carbon losses [16,65]. As in the case of forest degradation, few satellite-based estimates of secondary forest extent were available for Mato Grosso. Most studies estimated secondary forest area for only one period in time rather than following the dynamics of land abandonment and re-clearing of secondary forest. Previous estimates of the amount of historic deforestation in some stage of forest regrowth varied from 12-17% in Mato Grosso in three studies conducted with satellite data from 2000-2008 [66-68]. Across the entire Brazilian Amazon, the amount of historic deforestation in some stage of secondary forest regrowth ranged from 20-36% over different epochs [56,68,69]. However, a rigorous comparison of secondary forest data products was not possible due to differences in the timing of recent studies.

### 5.4 Forest carbon stocks

The amount and spatial distribution of forest carbon stocks in Amazonia are major sources of uncertainty in estimates of emissions from deforestation and forest degradation [22]. [70] estimated the total forest carbon storage in the Brazilian Amazon as 39-93 Pg C, but the seven data products reviewed in that study disagreed about the spatial distribution of low and high-biomass forest types within the region. Recent efforts to refine maps of forest carbon stocks in Amazonia have focused on new plot measurements of forest biomass [71], improved allometric relationships relating wood volume to biomass [72,73], and extrapolation of plot-based data using climate metrics [74], satellite-based estimates of forest canopy reflectance [75], and geostatistical methods [76]. Revised estimates of the total forest carbon stocks in Amazonia fall within the original range described by [70], albeit with lower forest biomass in areas of active deforestation in southern and eastern Amazonia than previously estimated [19,73,75]. Remaining uncertainties in the spatial distribution of forest biomass arise from the small number of forest plots [77] and the influence of historic land use on forest carbon stocks, especially along the deforestation frontier [16,49,70,78]. Direct estimates of aboveground biomass from LiDAR or Radar remote sensing instruments have the potential to address these concerns [40-42,79], but no direct satellite-based measurements of forest biomass were available for this study.

We compared one Tier 1 and five Tier 2 datasets of forest carbon stocks in Mato Grosso (Table 2). Tier 1 data for carbon stocks in tropical forests represent continental-scale averages for each forest type [53,80]. Tier 2 biomass datasets were derived from Amazon forest inventory plots, either from the Brazilian government's RADAMBRASIL survey [54] or a compilation of forest biomass plots from the scientific literature [70,75]. The RADAMBRASIL inventory is the most intensive survey of timber volumes in Brazilian forests conducted to date, with 440 one-hectare plots in Mato Grosso [54]. Converting timber volume into AGLB, including all plants regardless of timber utility, requires the use of a biomass conversion and expansion factor [18,73,81]. Similarly, aboveground dead biomass (AGDB) and belowground biomass (BGB) are typically estimated using relationships among field-measured AGLB, woody debris, and root-shoot ratios [70,72,73,82] (Table 2). Data products from Houghton and Saatchi *et al. *were based on forest biomass plots from the scientific literature, adjusting for AGDB and BGB in a similar manner when these quantities were not directly measured [70,75].

Tier 2 biomass maps for Amazonia rely on statistical methods to extrapolate plot-based measurements across the spatial extent of forest cover. Initial maps of forest biomass used simple interpolation between plot locations [70] or land cover information to assign average plot biomass values to each forest type [18,81]. Recently, additional variables such as climate, soils, topography, and forest phenology metrics derived from satellite data have been used to characterize forest biomass between plot locations [73-76]. We selected the most recent map product from each plot data source (RADAMBRASIL: Imazon, scientific literature: Saatchi *et al*.) for comparisons with Approach 3 deforestation data. The Imazon and Saatchi *et al. *data products represent substantial methodological advances over simple interpolation or forest type maps for estimating the spatial distribution of forest biomass in Amazonia [75,76]. These data products are labeled as Tier 2.m for 'map' in Table 3 to differentiate these spatially-explicit biomass maps at 1 km spatial resolution from spatially-averaged forest biomass data by forest type, state, or country (Tier 2.a for 'average'). Although both Tier 2.m data products include internal estimates of map accuracy based on cross-validation techniques, neither Saatchi *et al. *nor Imazon data products have been rigorously validated using independent estimates of contemporary forest carbon stocks. Therefore, all Tier 2 data products (2.m and 2.a) were treated equally in our summary of data products according to Tier/Approach.

## 6. List of abbreviations

AGDB: Aboveground Dead Biomass; AGLB: Aboveground Live Biomass; BGB: Below Ground Biomass; Imazon: Instituto do Homem e Meio Ambiente da Amazônia; INPE: Instituto Nacional de Pesquisas Espaciais; IPCC: Intergovernmental Panel on Climate Change; PRODES: Program for the Annual Estimation of Deforestation in the Amazon; REDD+: Reducing Emissions from Deforestation and forest Degradation and enhancing forest carbon stocks through conservation and sustainable forest management; SEMA: Secretaria Estadual do Meio Ambiente; UNFCCC: United Nations Framework Convention on Climate Change.

## 7. Competing interests

The authors declare that they have no competing interests.

## 8. Authors' contributions

DCM, CMS, and BG designed the study. DCM conducted the review and analysis of deforestation and forest carbon stock data and drafted the manuscript. MHS, CMS, and BG were contributing authors. All authors have read and approved the final manuscript.

## 9. Authors' information

^1^NASA Goddard Space Flight Center, Greenbelt MD USA, douglas.morton@nasa.gov

^2^Instituto do Homem e Meio Ambiente da Amazônia (Imazon), Belém, PA, Brazil, marcio@imazon.org.br; souzajr@imazon.org.br

^3^The Nature Conservancy, Arlington, VA USA, bgriscom@tnc.org
